# Supplementary material for: WordCommentsAnalyzer: A windows software tool for qualitative research
Source: F1000Res. 2018 Sep 4;7:536. Originally published 2018 May 3. [Version 2] doi: 10.12688/f1000research.14819.2 (PMC6137411; doi:10.12688/f1000research.14819.2)
Supplement: Supplementary file 3 [file f1000research-7-17614-s0002.tgz › b2a79496-4ada-47c4-9682-a99ce867836d.zip!/Eight Iranian Health Journals/Journal of Research in Rehabilitation Sciences _14.docx]

© Journal of Research in Rehabilitation Sciences . This work is licensed under CC BY-NC 4.0 (https://creativecommons.org/licenses/by-nc/4.0/).

Volume & Issue: دوره 11، شماره 3: 1394

Title: تأثیر تغییر ارتفاع پاشنه کفش بر ثبات وضعیت زنان جوان سالم

http://jrrs.mui.ac.ir/index.php/jrrs/article/view/2328

مقدمه: افزایش ارتفاع پاشنه کفش می‌تواند با تغییر در زوایای مفاصل اندام تحتانی و ستون فقرات و الگوهای فعالیت عضلات، چالشی برای حفظ ثبات بدن محسوب شود. هدف از انجام این مطالعه، بررسی اثرات تغییر ارتفاع پاشنه کفش بر نوسانات مرکز فشار در خانم‌های جوان سالم بود. مواد و روش‌ها: در مطالعه بالینی حاضر که از نوع متقاطع- تصادفی بود، 32 دانشجوی دختر با میانگین سنی 4/1 ± 3/22 سال به صورت داوطلبانه شرکت کردند. متغیرهای نوسان مرکز فشار بدن در وضعیت ایستاده توسط صفحه نیرو و در چهار حالت تصادفی پای برهنه، کفش بدون پاشنه، کفش پاشنه 3 سانتی‌متر و کفش پاشنه 6 سانتی‌متری اندازه‌گیری شد و داده‌های به دست آمده با Repeated measures ANOVA، مورد تجزیه و تحلیل قرار گرفت. یافته‌ها: تحلیل آماری تغییرات معنی‌داری را در داده‌های دامنه جابه‌جایی و سرعت مرکز فشار طولی و جانبی بدن در مقایسه بین ارتفاع‌های مختلف پاشنه نشان داد (050/0 > P). بلند شدن ارتفاع پاشنه موجب افزایش میانگین سرعت (010/0 > P، 6/4 = (29و3)F)، طول جابه‌جایی (001/0 > P، 23 = (29و3)F) و دامنه نوسانات (001/0 > P، 5/11 = (29و3)F) مرکز فشار در جهت طولی بدن شد. نتیجه‎گیری: به نظر می‌رسد افزایش ارتفاع پاشنه باعث تغییرات در متغیرهای تعادلی ثبات وضعیتی بدن می‌شود و می‌تواند زمینه‌ساز کاهش تعادل باشد.

Title: تأثیر فوری استفاده از کفی Arch support بر مؤلفه‌های نیروهای عکس‌العمل زمین هنگام راه رفتن در افراد سالم

http://jrrs.mui.ac.ir/index.php/jrrs/article/view/2297

مقدمه: سنجش نیروهای عکس‌العمل زمین و سایر شاخص‌های کینتیکی راه رفتن، از ارزش کلینیکی برخوردار است. نوع کفش و کفی در تعامل با این عوامل است. هدف از انجام پژوهش حاضر، بررسی اثر فوری کفی‌های Arch support بر قله نیروهای عکس‌العمل زمین و زمان رسیدن به آن‌ها، ایمپالس، نرخ بارگذاری و گشتاور آزاد در مرحله استقرار راه رفتن در افراد سالم بود. مواد و روش‌ها: 16 مرد سالم با میانگین سنی 8/4 ± 4/29 سال، میانگین وزنی 6/12 ± 9/77 کیلوگرم و میانگین قد 8/5 ± 5/176 سانتی‌متر در این مطالعه شرکت نمودند. با استفاده از دو صفحه نیرو (1000 هرتز)، مؤلفه‌های نیروی عکس‌العمل زمین طی فاز استقرار راه رفتن در دو شرایط با و بدون استفاده از کفی اندازه‌گیری شد. سپس متغیرهای قله نیروهای عکس‌العمل زمین و زمان رسیدن به آن‌ها، ایمپالس، نرخ بارگذاری و گشتاور آزاد استخراج شد. آزمون آماری Repeated measures ANOVA در سطح معنی‌داری 05/0 ˂ P جهت تحلیل آماری مورد استفاده قرار گرفت. یافته‌ها: پوشیدن کفی موجب کاهش قله مؤلفه عمودی عکس‌العمل زمین در لحظه تماس پاشنه با زمین (9/6 درصد وزن بدن، 001/ 0‌= ‌P) و نرخ بارگذاری عمودی (4/8 درصد، 020/ 0‌=‌P ) شد، اما نیروی جلو برنده (7 درصد نیوتن بر وزن بدن، 001/ 0‌= ‌P) و ایمپالس قدامی- خلفی (2/1 درصد وزن بدن در ثانیه، 003/ 0‌= ‌P) و ایمپالس عمودی (2 درصد وزن بدن در ثانیه، 032/ 0‌=‌P ) را افزایش داد. نتیجه‎گیری: کاهش نیروی عکس‌العمل زمین و نرخ بارگذاری به وسیله کفی ممکن است خطر آسیب‌های اندام تحتانی ناشی از ایمپالس هنگام راه رفتن را کاهش دهد. استفاده از کفی Arch support می‌تواند با کاهش نیروهای وارد آمده بر بدن، از بروز آسیب‌های ورزشی جلوگیری کند.

Title: اثر تمرین شناختی بر کارایی شبکه کنترل اجرایی توجه و دقت پاسخ حرکتی

http://jrrs.mui.ac.ir/index.php/jrrs/article/view/2155

مقدمه: هدف از انجام تحقیق حاضر، بررسی تأثیر تمرین شناختی با تکالیف حافظه کاری بر کارایی شبکه عصبی کنترل اجرایی توجه و دقت پاسخ حرکتی بود. مواد و روش‌ها: این پژوهش از نوع نیمه تجربی بود و با طرح پیش‌آزمون و پس‌آزمون انجام شد. 20 دانشجوی دختر در تحقیق شرکت داشتند. گروه شناختی (10 نفر) تمرینات تکالیف حافظه کاری بینایی- فضایی را دریافت کردند و گروه شاهد (10 نفر) تمرینات ساده دو مهارت حرکتی بدون عامل شناختی را انجام دادند. پیش و پس از هشت جلسه تمرینی 45 دقیقه‌ای، از شرکت کنندگان آزمون شبکه‌های توجه برای سنجش کنترل اجرایی توجه و دقت پاسخ به دو مهارت حرکتی به عمل آمد. داده‌ها با استفاده از روش آماری تحلیل واریانس مکرر و نرم‌افزار SPSS در سطح معنی‌داری 05/0 مورد تحلیل قرار گرفت. یافته‌ها: آزمون تحلیل واریانس مکرر دو در دو تفاوت معنی‌داری را بین دو گروه در هر سه عامل (کنترل اجرایی توجه، دقت پاسخ مهارت فورهند و دقت پاسخ مهارت بک‌هند) نشان داد. گروه شناختی در عامل کنترل اجرایی توجه بهبود در کارایی شبکه را نشان داد؛ در حالی که در گروه شاهد تغییری مشاهده نشد. در عامل دقت پاسخ در گروه شناختی بهبود بیشتری با توجه به نمرات میانگین نسبت به گروه شاهد مشاهده شد.نتیجه‎گیری: با توجه به نتایج پژوهش، به نظر می‌رسد تمرین شناختی روی کارایی شبکه عصبی و دقت پاسخ حرکتی تأثیر مثبتی دارد و این موضوع نشان دهنده اثر افزایش یافته ارتباطات درون شبکه‌ای و انعطاف‌پذیری مغز به تکرار و تمرین است. بنابراین می‌توان گفت تمرینات شناختی به دلیل درگیری توانایی‌های شناختی بیشتر مانند حافظه، توجه و ادراک و همچنین درگیری بیشتر شبکه‌های مغزی و تسهیل اتصال سیناپسی، اثرات مثبت‌تری بر توانایی‌های شناختی و اجرا دارد

Title: بررسی تأثیر گوه خارجی و گوه خارجی بافت‌دار بر تعادل استاتیک در افراد مبتلا به بی‌ثباتی عملکردی مچ پا

http://jrrs.mui.ac.ir/index.php/jrrs/article/view/2009

مقدمه: بی‌ثباتی عملکردی به عنوان احساس خالی کردن مچ پا شرح داده می‌شود. نقص در حفظ تعادل یکی از اختلالات حسی- حرکتی مشاهده شده در افراد مبتلا به بی‌ثباتی عملکردی می‌باشد که علت آن، آسیب گیرنده‌های آوران موجود در لیگامان‌ها و کپسول مفصلی ذکر شده است. ارتز پا از طریق افزایش ورودی‌های حسی از سطح پلانتار پا و همچنین جلوگیری از ایجاد گشتاور نامناسب در اطراف مفصل مچ پا می‌تواند موجب بهبود تعادل شود. از این‌رو هدف مطالعه حاضر بررسی تأثیر گوه خارجی و گوه خارجی بافت‌دار بر تعادل استاتیک در بیماران مبتلا به بی‌ثباتی عملکردی مچ پا بود. مواد و روش‌ها: 20 ورزشکار با میانگین سنی 77/4 ± 57/25 سال و با تشخیص بی‌ثباتی عملکردی یک‌طرفه مچ پا وارد مطالعه شدند. اندازه‌گیری‌ها طی سه وضعیت با استفاده از دستگاه صفحه نیرو انجام گرفت. میانگین جابه‌جایی مرکز فشار، میانگین سرعت جابه‌جایی آن، مساحت ناحیه دایره‌ای محاط بر محدوده حرکت مرکز فشار (با اطمینان 95 درصد) و مساحت ناحیه چرخش مرکز فشار در واحد زمان به عنوان متغیرهای اندازه‌گیری تعادل محاسبه شد. نتایج با استفاده از آزمون‌های ناپارامتریک Friedman و Wilcoxon تحلیل گردید. یافته‌ها: استفاده از ارتزهای پا باعث کاهش میزان متغیرهای میانگین جابه‌جایی کلی و قدامی- خلفی مرکز فشار، مساحت ناحیه دایره‌ای محاط بر محدوده حرکت مرکز فشار (با اطمینان 95 درصد) و افزایش متغیرهای میانگین جابه‌‌جایی داخلی-خارجی مرکز فشار، سرعت جابه‌جایی داخلی- خارجی مرکز فشار و مساحت ناحیه چرخش مرکز فشار در واحد زمان شد، اما هیچ کدام از این تفاوت‌ها معنی‌دار نبود. نتیجه‎گیری: ارتزهای پا تغییر عمده‌ای در ثبات وضعیتی افراد دچار بی‌ثباتی عملکردی مچ پا ایجاد نکرد.

Title: بررسی تأثیر وضعیت چشم بر پتانسیل عضلانی برانگیخته دهلیزی

http://jrrs.mui.ac.ir/index.php/jrrs/article/view/1698

قدمه: پتانسیل عضلانی برانگیخته دهلیزی، آزمون مفیدی جهت بررسی عملکرد عصب دهلیزی تحتانی و ساکول می‌باشد. دامنه پتانسیل عضلانی برانگیخته دهلیزی متناسب با سطح فعالیت الکترومیوگرافی زمینه است. در عضله پشت گوشی، مقدار الکترومیوگرافی با وضعیت چشم به طور مستقیم مرتبط می‌باشد. بنابراین پژوهش حاضر با هدف بررسی امکان تأثیر وضعیت چشم بر پتانسیل عضلانی برانگیخته دهلیزی صورت گرفت. مواد و روش‌ها: در این مطالعه مقطعی- مقایسه‌ای، پتانسیل عضلانی برانگیخته دهلیزی در 30 فرد هنجار محدوده سنی 30-18 سال با ارایه محرک تن برست 500 هرتز با شدت 95 دسی‌بل nHL در سه وضعیت چشم شامل چشم‌ها در جهت موافق با چرخش سر، چشم‌ها روبه‌رو و چشم‌ها در جهت مخالف با چرخش سر در هر دو گوش ثبت شد. یافته‌ها: در هر دو گوش اختلاف معنی‌داری بین میانگین دامنه هر سه وضعیت چشم وجود داشت (05/0 > P). در هر دو گوش، میانگین دامنه پتانسیل عضلانی برانگیخته دهلیزی در وضعیت چشم‌ها مخالف با چرخش سر، کمتر و در وضعیت چشم‌ها موافق با چرخش سر، بیشتراز دو وضعیت دیگر گردید (05/0 > P). در هر سه وضعیت چشم و در هر دو گوش، میانگین زمان نهفتگي امواج P13 و N23 هیچ گونه اختلاف معنی‌داری نشان نداد (05/0 < P).نتیجه‎گیری: نتایج مطالعه حاضر نشان می‌دهد که میزان پاسخ بسته به وضعیت چشم فرد حین ثبت پتانسیل عضلانی برانگیخته دهلیزی تغییر می‌نماید. بنابراین کنترل وضعیت چشم حین ثبت آزمون توصیه می‌شود.

Title: بررسی تأثیر فوری کفش‌های غلتکی بر پوسچر ‏تنه و سر و گردن زنان سالم در حالت ایستاده

http://jrrs.mui.ac.ir/index.php/jrrs/article/view/2241

مقدمه: استفاده از کفش‌های غلتکی با هدف بهبود توانایی راه رفتن توصیه می‌شود. با توجه به تغییرات گزارش شده در پوسچر و کینماتیک مفاصل مچ پا، زانو و لگن، یکی از مهم‌ترین فرضیات متخصصان بالینی در تجویز این نوع کفش، تغییر پوسچر تنه و سر و گردن می‌باشد. هدف از انجام این مطالعه، بررسی تأثیر فوری یک نوع کفش غلتکی بر پوسچر تنه و سر و گردن حین ایستادن در زنان سالم بود. مواد و روش‌ها: مطالعه حاضر بر روی 10 زن سالم با میانگین سنی 8/1 ± 5/24 سال انجام گرفت. موقعیت سه بعدی 11 مارکر بازتابنده نور که بر روی ستون فقرات و پیشانی نصب شده بود، توسط 8 دوربین 100 هرتزی، حین ایستادن در شرایط پا برهنه و پوشیدن یک نوع کفش غلتکی ثبت گردید. شرایط به صورت تصادفی انتخاب و ثبت در هر کدام از شرایط سه مرتبه تکرار شد. به منظور ارزیابی پوسچر در صفحه ساجیتال، شعاع قوس لومبار، قوس توراسیک و زاویه بین سر و گردن با صفحه افق مورد محاسبه قرار گرفت. برای مقایسه آماری دو حالت، آزمون Paired t در سطح معنی‌داری 05/0 مورد استفاده قرار گرفت. یافته‌ها: ایستادن با کفش غلتکی در مقایسه با حالت پا برهنه، شعاع قوس لومبار را به صورت معنی‌دار و به میزان 23/11 درصد کاهش داد (006/0 = P). شعاع قوس توراسیک به میزان 45/4 درصد افزایش یافت؛ اگرچه این تغییر معنی‌دار نبود (460/0 = P). همچنین، تغییر معنی‌داری در زاویه بین سر و گردن و صفحه افق مشاهده نشد (470/0 = P). نتیجه‎گیری: نتایج مطالعه حاضر نشان دهنده ایجاد پوسچر اکستانسوری لومبار در وضعیت ایستاده به دنبال پوشیدن کفش‌های غلتکی می‌باشد. بنابراین، کفش‌های غلتکی شاید بتواند به عنوان مداخله درمانی در مواردی که نیاز به ایجاد پوسچر اکستانسوری ناحیه لومبار است، مورد استفاده قرار گیرد.

Title: تاثیر تمرینات طناب زنی بر قوس کف پای دانش آموزان پسر 13-10 ساله دارای کف پای صاف، گود و طبیعی

http://jrrs.mui.ac.ir/index.php/jrrs/article/view/2398

مقدمه: ﻗوس کف پا پا ﺳﺎزه ﻣﻌﻤﺎري استﻛﻪ ﺗﻤﺎﻣﻲ اﺟزاي ﭘﺎ ﻳﻌﻨﻲ ﻣﻔﺎﺻل، ﻟﻴﮕﺎﻣﻨتﻫﺎ و ﻋﻀﻼت را داﺧل ﻳک سیستم واﺣد  تلفیق‫ کرده و ازبخش­هاي بسیار فعال در فعالیت­های جهشی مانند طناب­زنی می­باشد. این تحقیق در پی بررسی تاثیر12هفته تمرینات طناب­زنی بر قوس کف پای دانش­آموزان پسر 13-10ساله دارای کف­پای صاف، گود و طبیعی است. روش ها: در این مطالعه نیمه تجربی تعداد 90 دانش آموز پسر 10 تا 13 ساله بخش جرقویه سفلی به صورت هدفمند در دسترس به عنوان نمونه انتخاب و در سه گروه کف پای گود، صاف و طبیعی، هرگروه 30 نفر قرار گرفتند. سپس آزمودنی های هر گروه به صورت تصادفی به دو گروه تجربی و کنترل هرگروه 15 نفر تقسیم شدند. جهت ارزیابی اولیه از جعبه آینه و سپس برای مشخص کردن ساختار پا از اسکنر کف پا استفاده شد و قوس كف­پاي آنها با شاخص استاهلي(Staheli index) به صورت كمي طبقه­بندي گردید. آزمودنی های گروه های تجربی به مدت 12 هفته،هر هفته سه جلسه 45 دقیقه ای در یک پروتکل تمرینی مشترک شرکت کردند. در طی این مدت آزمودنی­های گروه کنترل در هیچ برنامه فعالیت بدنی منظمی شرکت نکردند. جهت تجزيه و تحليل آماري داده­ها ازANOVA  براي داده­هاي تكراري در بسته آماری SPSS ویرایش22 استفاده شد(05/0p<). نتایج: تغييرات درون گروهي(پس آزمون نسبت به پيش آزمون) و همچنين روند اين تغييرات (شيب خط تغييرات)تنها در متغیرهای مربوط به گروه کف پای صاف معنا دار بود(005/0p<). تفاوت بین گروهی در هیچ یک از گروه­ها معنادار نبود. نتیجه گیری: به نظر می­رسد تمرینات طناب­زنی باعث بهبود قوس کف پا در افراد دارای کف­پای صاف منعطف می گردد.

Title: بررسی تأثیر مداخله حافظه فعال واج‌شناختی بر کاهش میزان شدت لکنت کودکان پیش دبستانی: یک پژوهش مورد منفرد

http://jrrs.mui.ac.ir/index.php/jrrs/article/view/2320

مقدمه: حافظه فعال به عنوان یکی از اجزای تولید گفتار مطرح است و نقص در آن می‌تواند منجر به اختلالات گفتار و زبان شود. مطالعات زیادی بیان کرده‌اند که كودكان مبتلا به لكنت نسبت به كودكان طبيعي، نقايصي در زمينه حافظه فعال نشان مي‌دهند. هدف از مطالعه حاضر، بررسی تأثیر مداخله حافظه فعال واجی بر کاهش میزان شدت لکنت کودکان پیش دبستانی بود. مواد و روش‌ها: این مطالعه به شیوه پژوهش مورد منفرد، بر روی چهار کودک 5 تا 6 ساله مبتلا به لکنت انجام گردید. این کودکان در یک برنامه مداخله تکرار ناکلمه که 18 جلسه و به مدت 6 هفته به طول انجامید و به صورت مورد منفرد (مدل AB) انجام شد، شرکت کردند. برای تحلیل داده‌ها از روش تحلیل ديداري و اندازه اثر Cohen's d استفاده شد. یافته‌ها: تجزیه و تحلیل داده‌ها نشان‌ داد که به دنبال مداخله، تغییرات معنی‌داری به صورت کاهش درصد هجاهای لکنت شده در شرکت ‌کنندگان مشاهده شد. نتیجه‎گیری: نتایج مطالعه حاضر نشان ‌دهنده تأثیر مداخله حافظه فعال واجی بر کاهش میزان شدت لکنت در کودکان مورد مطالعه است و شواهد اولیه را در جهت به کارگیری روش‌های درمانی سایکولینگویستیک برای درمان کودکان مبتلا به لکنت فراهم می‌آورد.

Title: تأثیر تمرینات ثبات دهنده تنه بر تعادل پویای ورزشکاران مبتلا به پیچ خوردگی مزمن مچ پا

http://jrrs.mui.ac.ir/index.php/jrrs/article/view/2248

مقدمه: پیچ خوردگی مچ پا از رایج‌ترین آسیب‌های ورزشی است که به دنبال آن تعادل ورزشکاران را دچار اختلال می‌نماید. این مطالعه با هدف بررسی تأثیر شش هفته تمرینات ثبات دهنده تنه بر تعادل پویای ورزشکاران مبتلا به پیچ خوردگی مزمن مچ پا انجام گرفت. مواد و روش‌ها: مطالعه حاضر از نوع کارآزمایی بالینی یک سوکور با دو گروه مداخله و شاهد بود. تعداد 30 نفر ورزشکار مبتلا به پیچ خوردگی مزمن مچ پا به طور تصادفی در دو گروه مداخله و شاهد قرار گرفتند. در گروه شاهد تنها فیزیوتراپی رایج و در گروه مداخله علاوه بر فیزیوتراپی رایج، تمرینات ثبات دهنده تنه نیز به کار گرفته شد. قبل و بلافاصله بعد از انجام مداخلات، تعادل پویای این افراد با استفاده از آزمون تعادلی ستاره در هشت جهت توسط آزمونگر بررسی شد. داده‌های به دست آمده با استفاده از آزمون‌های آماری 2c، Independent t، Paired t و تحلیل کوواریانس در نرم‌افزار SPSS در سطح معنی‌داری 05/0 مورد تجزیه و تحلیل قرار گرفت. یافته‌ها: پس از کنترل اثر پیش‌آزمون، امتیازات مربوط به آزمون تعادلی ستاره در هر هشت جهت در گروه مداخله، در مقایسه با گروه شاهد بهبودی معنی‌داری داشت (05/0 > P). نتیجه‎گیری: انجام تمرینات ثبات دهنده تنه نسبت به فیزیوتراپی رایج، بهبود معنی‌داری را در تعادل پویای ورزشکارن مبتلا به پیچ خوردگی مزمن مچ پا ایجاد می‌نماید، بنابراین پیشنهاد می‌شود که تمرینات ثبات دهنده در کنار فیزیوتراپی رایج در توان‌بخشی این افراد مورد توجه قرار گیرد.

Title: تغییرات عضله اسکلتی متعاقب اسپاستیسیتی

http://jrrs.mui.ac.ir/index.php/jrrs/article/view/2088

اسپاستیسیتی یک عارضه نورولوژی است که ثانویه به ضایعه نورون محرکه فوقانی ایجاد می‌شود و اثر مهمی بر روی عضله اسکلتی دارد. ضایعه نورون محرکه فوقانی ممکن است به عللی همچون سکته مغزی، ضربه مغزی، ضایعه نخاعی یا بیماری‌های دژنراتیو مانند مولتیپل اسکلروزیس یا ضایعات مغزی دوران بارداری مانند فلج مغزی ایجاد گردد. توانایی عملکردی این بیماران در اثر اسپاستیسیتی به طور شدیدی به مخاطره می‌افتد، اما مکانیسم‌های پایه این عارضه هنوز به طور واضح مشخص نشده است. در این مقاله مروری شواهد و مطالعات جدید در مورد تغییرات بافت عضله اسکلتی متعاقب شرایط اسپاستیک مورد بررسی قرار گرفت. تحقیقات تجربی مورد مطالعه شامل طیف متفاوتی از روش‌هایی بودند که مکانیک مفصل، مکانیک بافت و شکل عضله را مورد مطالعه قرار دادند.
